# Prevalence, Incidence, Influence Factors, and Cognitive Characteristics of Amnestic Mild Cognitive Impairment Among Older Adult: A 1-Year Follow-Up Study in China

**DOI:** 10.3389/fpsyt.2020.00075

**Published:** 2020-02-28

**Authors:** Wei Li, Lin Sun, Shifu Xiao

**Affiliations:** ^1^ Department of Geriatric Psychiatry, Shanghai Mental Health Center, Shanghai Jiao Tong University School of Medicine, Shanghai, China; ^2^ Alzheimer’s Disease and Related Disorders Center, Shanghai Jiao Tong University, Shanghai, China

**Keywords:** Chinese, amnestic mild cognitive impairment, prevalence, incidence, executive function

## Abstract

**Background:**

The risk and protective factors of amnestic mild cognitive impairment (aMCI) and its prevalence as well as incidence among old adult in Chinese community are still unclear.

**Methods:**

We carried out this 1-year longitudinal study to survey a random sample of 3,246 community elders aged 60 and over in China. All subjects were required to complete a comprehensive clinical assessment, physical examination and several neuropsychological tests at baseline and follow-up. What’s more, we also collected their lifestyle information by a standardized questionnaire.

**Results:**

We found that the prevalence of aMCI was 17.1%, while the incidence of aMCI among Chinese old adult was 70.57 per 1,000 person-years. By using Cox regression analysis, we found that male sex (p = 0.001, OR = 0.489, 95%CI 0.319~0.751) and reading (p = 0.023, OR = 0.533, 95%CI 0.310~0.917) were protective factors for against aMCI. Old adult who developed aMCI in the future showed multiple cognitive impairments (such as immediate memory, associative learning memory and executive function) in their early stage, and Wechsler’s Block Design (p = 0.027, OR = 0.969, 95%CL 0.943~0.996) could predict whether subjects would turn into aMCI in the future.

**Conclusions:**

The present study suggests that aMCI is a considerable health problem in China. Executive dysfunction may be an indicator of future development of aMCI in the old normal adult.

## Introduction 

Mild cognitive impairment (MCI) is an intermediate phase between normal ageing and early dementia, and is associated with increased risk of Aizheimer's disease (AD) (1). MCI is characterized by cognition impairment, especially in memory, but has no significant impact on daily life (2). MCI can be further divided into four subtypes: amnestic MCI-single domain (sa-MCI), amnestic MCI-multiple domains (ma-MCI), nonamnestic MCI-single domain (sna-MCI), and nonamnestic MCI-multiple domains (mna-MCI) (3), among which, amnestic mild cognitive impairment (including sa-MCI and ma-MCI), characterized by diminished delayed free recall ability, is the subtype most correlated with AD (4). And the autopsy results show that the pathological characteristic of aMCI is consistent with the stage preceding AD (5). According to previous studies, 10–15% of aMCI will be converted to AD (6), while the nonamnestic MCI will develop into dementia with Lewy bodies or frontotemporal dementia (7).

Determining the MCI and subtype prevalence is essential to develop preventive approaches for old adult (5). However, at present, the prevalence of MCI is difficult to calculate, as it depends on the precise diagnostic criteria (8). For example, a systematic review reports that the prevalence of MCI ranges widely from 0.5% to 42% (5). Nevertheless, since AD increases double every 5 years after age 65 (9), it is important to evaluate adults with aMCI from several years before that age. So far, there are only two studies involving the prevalence of aMCI in China, Our previous study found that the prevalence of aMCI among old adult in Shanghai community was 22.3%, and the incidence (per 1,000 person-years) was 96.9 (10), while another study carried out in Tianjin showed that the prevalence of MCI was 11.33% (11). So our conclusions are not consistent, and this may be due to different inclusion criteria (we included subjects aged 60 and over, and their subjects aged 65 and over) and assessment tools (we used MOCA to assess the global cognitive function of the subjects, while they used MMSE, and the greater sensitivity of the MOCA (vs MMSE) might contribute to a higher rate of aMCI in Shanghai).

Due to the inconsistent conclusions and regional differences in the previous studies, we conducted a 1-year follow-up study across the country [including 20 target communities (18 urban and 2 rural) located in the eastern, mid, and western parts of China] to explore the prevalence, incidence, influence factors as well as cognitive characteristics of aMCI among the old adult in Chinese communities. The 2014 World Alzheimer Report pointed out that low education in early life, hypertension in midlife as well as diabetes and smoking across the life course were associated with dementia (12), therefore, we speculate that the above factors may also affect the aMCI.

## Materials and Methods

### Calculation of Sample Size

Accumulated evidence suggests that the prevalence of MCI (Pexp) is between 10% and 20% (10, 13, 14). Based on the sample size calculation formula: N = (1.96/d)^2^×(Pexp) ×(1-Pexp), (d = ± 3%), we determined that the sample size for this study should be no less than 785 (15).

### Participants

This follow-up study was a collaborative effort of 15 institutions located in the eastern, middle and western parts of China, which was conducted from March 2011 to July 2012. Each institution identified one or more target communities, and 20 target communities (18 urban and 2 rural) entered in this project. Finally, a total of 15,304 individuals, aged 60 and over, registered in the 2010 national census as permanent residents of these communities, were put into a database. The inclusion criteria were as follows: (1) 60 years and older; (2) permanent residents; (3) without evidence of serious physical illness, such as cancer and acute myocardial infarction; (4) without serious mental illness, such as mental retardation and schizophrenia; and (5) agreed to participate in the study. Exclusion criteria were as follows: (1) less than 60 years old; (2) external population; (3) acute stress state; (4) serious physical illness or mental illness; (5) refused to participate in the study; Then, we randomly selected 3,246 old adult people as the potential participants (among these selected, 111 participants were excluded for incomplete data, and there was no bias due to missing data). Among these groups, illiteracy accounted for 17.1%, primary education accounted for 25.5%, junior high school accounted for 24.7%, senior high school or technical secondary school accounted for 15.7%, junior college accounted for 6.4%, university or above accounted for 10.6%. And the sampling process has been outlined in detail in our previous studies (1).

### Aims of the Project

- Estimate the prevalence of aMCI among the old adult in Chinese communities.
- Develop a screening program for aMCI that uses a mathematical algorithm which integrates both biological and psychological measures.
- Implement screening procedures in a consultative network including psychiatrists, clinical psychologists and community physicians to determine the sensitivity and specificity of the algorithm for identifying prodromal cases of aMCI.
- Develop China-specific, standardized treatment protocols for non-pharmacological treatment of aMCI (using cognitive training).
- Establish norms for brain volumes and other measures obtained from magnetic resonance imaging (MRI) as well as a bank of biological samples.

Ethical approval was issued by Shanghai Mental Health Centre, and all the participants had signed an informed consent before the study was initiated.

### Clinical Assessment

#### Amnesic Mild Cognitive Impairment

The diagnosis of MCI was based on the diagnosis standard of Petersen (16): (1) self- or informant -reported cognitive complain; (2) objective memory impairment; (3) preserved independence in functional abilities; and (4) absence of dementia. And if people with MCI performed poorly in episodic memory, they were considered as amnestic MCI (aMCI), while if they performed poorly in other cognitive areas rather than memory, they were considered as non-amnestic MCI (naMCI) (17).

#### Subjective Memory Decline

The diagnosis of subjective memory decline (SCD) was based on a conceptual framework of criteria for identification of SCD (18): (1)self -reported cognitive decline; 2 the onset age was more than 60 years old; (2) the presence of subjective memory decline had persisted for ≥6 months; (3) objective cognitive score in normal range.

#### Dementia

The diagnosis of dementia was based on the Diagnostic and Statistical Manual of mental disorders, Fourth Edition (DSM-IV) (19). When dementia was diagnosed, participants would be further classified into three subtypes: Alzheimer disease (AD), vascular dementia (VD), and mixed dementia (MD). Diagnostic criteria for AD were based on the criteria issued by the National Institute of Neurological and Communicative Disorders and Stroke–Alzheimer's Disease and Related Disorders Association (20). Diagnostic criteria for VD were based on the reports of the NINDS-AIREN International Workshop (21). And MD included other types of dementia (such as frontotemporal dementia, alcoholic dementia, dementia with Lewy bodies, Parkinson's disease with dementia, etc) that could not be diagnosed.

#### Cognitively Unimpaired (CU)

Participants were considered to be cognitively unimpaired if there were: (a) without subjective memory or other cognitive complaints; (b) without evidence by extensive clinical evaluation or history of memory or other cognitive decline; (c) global Clinical Dementia Rating Scale (CDR) (22) score of 0 rated by the clinician; (d) objective cognitive score in normal range (23).

### Neuropsychological Tests

#### Cognitive Assessment

A series of neuropsychological tests, including the Mini- mental State Examination (MMSE) (24), the Montreal Cognitive Assessment (MoCA) (25), Digit Span (26), Associative Learning Test (ALT), Visual Identification Test (VIT), Verbal Fluency (VF), Auditory Verbal Learning Test (AVLT) (27), Wechsler Adult Intelligence Scale (WAIS)-III Block Design, and Wechsler Adult Intelligence Scale (WAIS)-III picture completion were used to assess the cognitive function of subjects. And neuropsychiatric Inventory (NPI) (28) was used to evaluate psychiatric and somatic symptoms. The diagnosis of aMCI in the present study was characterized by MMSE scores higher than, or equal to, 25, 21, or 18 for participants who had a middle school or higher education, an elementary school education, or no education, respectively (29).

#### Quality of Life Assessment

The quality of life of participants will be assessed utilizing the Life Event Scale (LES), and the Social Support Rating Scale (SSRS) (1).

### MR Image Acquisition and Processing

Brain structure image was acquired by using a Siemens Magnetom Verio 3.0T scanner (Siemens, Munich, Germany). The parameters of T1-weighted 3D magnetization prepared rapid gradient echo (MPRAGE) sequences were as follows: TE = 2.98 ms, TR = 2,300 ms,; matrix size = 240 × 256; flip angle of 9 degree, field of view (FOV) = 240 × 256 mm; slice thickness = 1.2 mm. Volumetric data was assessed by automated procedures, which have been described by Wolz R et al. (30). For each subject, volume and asymmetry with various brain areas as well as the brain size index were extracted (by using FreeSurfer).

### Laboratory Tests

Peripheral blood samples (5 ml) were collected between 7 and 9 am after an overnight fast. A regular blood panel was carried out to measure hemoglobin, white blood cells, red blood cells, neutrophil granulocytes, mean cell volume, and platelets. Biochemical tests assessed bilirubin, creatinine, potassium, chloride, sodium, glucose, triglyceride, cholesterol, total proteins, alanine transaminase, aspartate transaminase, blood urea nitrogen, high density lipoprotein, low density lipoprotein, apolipoprotein A, apolipoprotein B, and lipoprotein. Separated serum and plasma (200 ul each) were stored in -70°C freezers.

**Figure 1:**
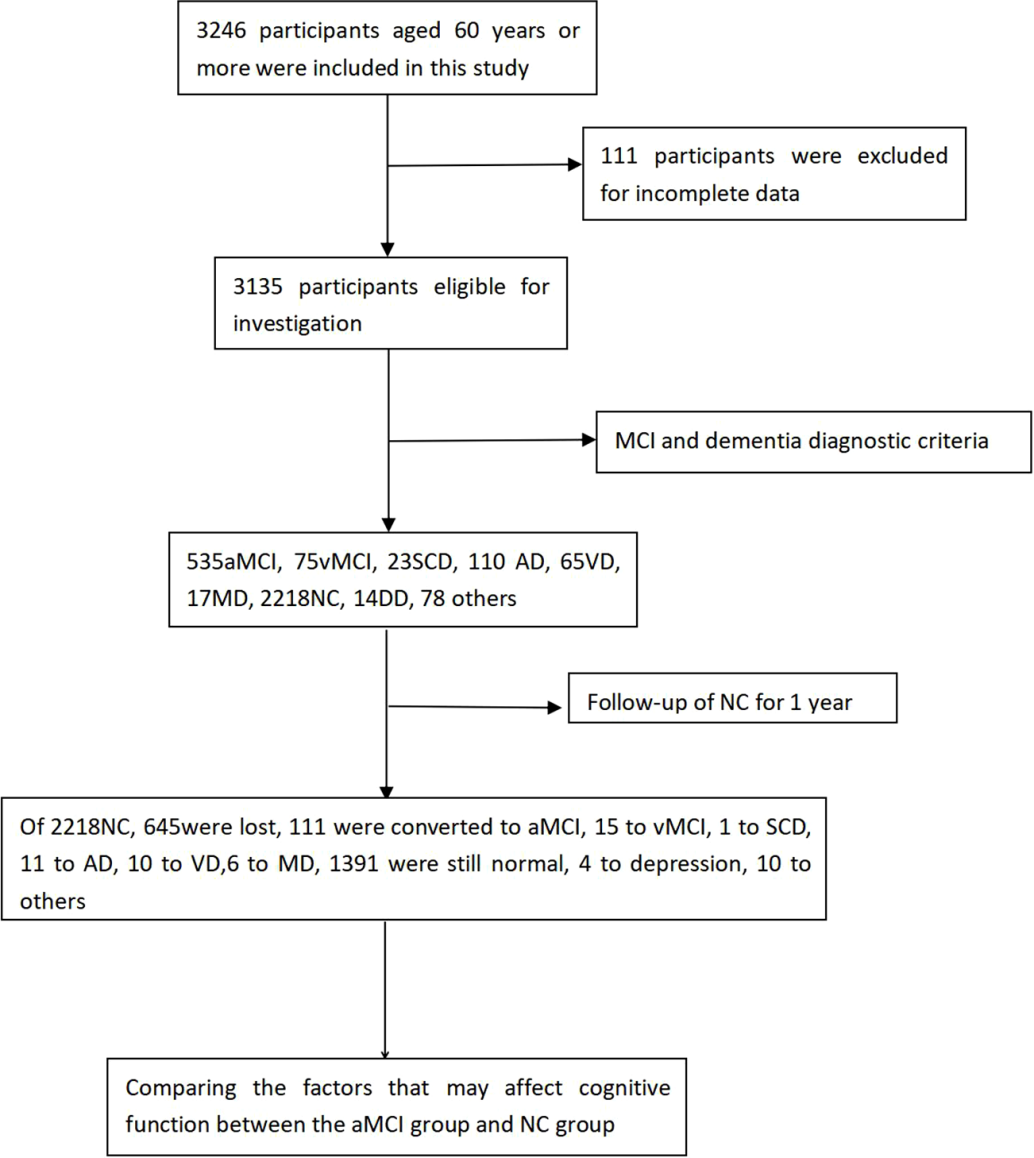
Flow diagram of the study population in the project. aMCI, vMCI, SCD, AD, VD, MD, NC, DD stand for amnestic mild cognitive impairment, Vascular mild cognitive impairment, Subjective cognitive impairment, Alzheimer disease, Vascular dementia, mixed dementia, normal control, depression, respectively.

All subjects were obliged to finish a baseline examination including a review of their medical history, physical and neurological examinations, neuropsychological tests, laboratory tests, and MRI scans. Based on clinical evaluation and neuropsychological test, 535 participants were diagnosed as aMCI, 75 as vascular MCI(vMCI), 23 as subjective cognitive impairment (SCD), 110 as Aizheimer's disease(AD), 65 as vascular dementia (VD), 17 as mixed dementia (MD), 2218 as cognitively unimpaired (CU), 14 as depression(DD), as well as 78 were unable to diagnose (such as anxiety, obsession, alcohol dependence, syphilis, and so on, due to the small proportion of the above diseases, we did not include them in the statistics). Next, we followed up 2,218 cognitively unimpaired aging people for 1 year, and repeated the baseline survey (follow-up evaluation). **Figure 1** describes our research process.

### Social Population Information

Social population information was gathered by self-reported. And the following data, such as name, age, gender, years of education, daily living information (smoking, drinking alcohol, drinking tea, taking exercise, hobby, reading, playing music, surfing the internet), and history of disease(depression, diabetes, and hypertension) were collected by standardized questionnaire. In terms of daily living information, taking reading as an example, participants were asked the following question “Do you read?” If the answer is “yes,” the participants were further asked to report their frequency of participation, such as “almost every day,” “sometimes,” or “never” (31). In the present study, we defined reading almost every day as reading.

## Statistical Analysis

The continuous variables were presented as mean± SD, while categorical variables were expressed as the frequency (%). The prevalence of aMCI was calculated by splitting the total number of cases diagnosed (n = 535) at baseline by the total number of participants (n = 3135, 111 persons were excluded for missing diagnoses). The incidence for aMCI (expressed as the number of cases per 1,000 person-years) was established by dividing the numbers of newly diagnosed cases at the 1-year follow-up visit. And the influence factors of aMCI were analyzed by Cox logistic regression (whether the cognitively unimpaired aging people changed into aMCI were taken as state variable and age as time variable). Finally, we randomly selected 111 cognitively unimpaired aging people who were not converted to aMCI (matched with age, sex, and education of those who were converted to aMCI) and compared the baseline and follow up neuropsychological tests between the two groups by using independent sample t-test (normally distributed data) or Mann-Whitney U(non-normally distributed data). Then, Cox regression analysis (111 pairs) was used to screen which neuropsychological test was most helpful to predict aMCI. And LSD-t test was used to correct multiple comparisons. Statistical analysis was performed by using SPSS version 22.0 and a p-value < 0.05 was regarded as significant.

## Results

### Characteristics of the Total Sample

A total of 3,246 nationally representative people (with 1,473 males, 45.4%), completed the survey. Their age range was 60–99, with an average age of 71.58 ± 8.061. Of them, 111 were excluded because of missing data. And there was no significant difference (p > 0.05) in age, gender and education between the excluded population and the study population.

### Characteristics of the Old Normal Adult Who Were Followed Up

2218 old adult people (1,074 males, age:70.14 ± 7.535) with cognitively unimpaired were followed up for one year. Among them, 645 (29.1%) lost their visits, 111(5.0%) changed into aMCI, 15(0.7%) into vMCI, 1(0.0) into SCD, 11 (0.5%) into AD, 10(0.5%) into VD, 6 (0.3%) into MD, 4(0.2%) into depression, 10 (0.5%) into others, while 1,391(62.7%) were still cognitively unimpaired (**Figure 1**). **Supplementary Table 1** shows the general demographic differences between the lost population and the follow-up population. Next, we compared the general demographic data of normal -normal group and normal-aMCI group, and we found that the average age (75.30 ± 7.123) of the normal-aMCI group was significantly higher than that (69.82 ± 7.336) of the normal-normal group, while the years of education (6.17 ± 5.500) of the normal-aMCI group were lower than that (9.83 ± 4.876) of the normal-normal group. What’s more, the normal-aMCI group had a higher proportion of depression, and a lower proportion of male, hobby, reading, playing music, and surfing the internet compared with the normal-normal group (p < 0.05). In addition, the volume of left and right hippocampus of the normal-aMCI group was lower (p < 0.05) than that of the normal-normal group. However, there was no significant difference (p > 0.05) between the two groups in NPI total score and subtest. **Table 1** presents the results.

**Table 1:** Demographic, health, and disease-related for Normal-aMCI and Normal-Normal group.

Characteristics	Normal-aMCI (N=111)	Normal-Normal (n=1,391)	t/ X^2^	P-value
Age, y	75.30 ± 7.123	69.82 ± 7.336	7.214	<0.001*
Education, y	6.17 ± 5.500	9.83 ± 4.876	-6.633	<0.001*
Male,n (%)	37(33.3)	694(49.9)	11.283	0.001*
Smoking,n (%)	27(24.3)	408(29.3)	1.253	0.279
Drinking alcohol,n (%)	16(14.4)	288(20.7)	2.519	0.140
Drinking tea,n (%)	50(45.0)	727(52.3)	2.146	0.167
Taking exercise,n (%)	80(72.1)	1,068(76.8)	1.264	0.295
Hobby,n (%)	53(5.4)	931(66.9)	16.742	<0.001*
Reading,n (%)	23(20.7)	477(34.3)	8.525	0.003*
Playing music, n (%)	17(15.3)	368(26.5)	6.693	0.009*
Surfing the internet, n (%)	6(5.4)	174(12.5)	4.918	0.023*
Depression, n (%)	10(9.0)	60(4.3)	5.101	0.033*
Diabetes, n (%)	14(12.6)	208(15.0)	0.447	0.580
Hypertension, n (%)	58(52.3)	646(46.4)	1.394	0.277
Right hippocampus, mm^3^	3453.37 ± 433.25	3,886.07 ± 458.32	-3.432	0.001*
Left hippocampus, mm^3^	3348.45 ± 331.05	3,653.17 ± 468.51	-3.126	0.017*
NPI	2.56 ± 7.087	2.24 ± 6.786	0.468	0.640
Delusion	1.81 ± 0.580	1.70 ± 0.711	1.884	0.062
Hallucination	1.82 ± 0.575	1.70 ± 0.710	1.686	0.092
Aggressive	1.82 ± 0.575	1.70 ± 0.710	1.686	0.092
Depression/dysthymia	1.77 ± 0.598	1.69 ± 0.712	1.260	0.208
Anxiety	1.79 ± 0.590	1.69 ± 0.712	1.426	0.154
Euphoria	1.82 ± 0.575	1.70 ± 0.710	1.667	0.096
Apathy	1.82 ± 0.575	1.71 ± 0.869	1.241	0.215
Disinhibition	1.82 ± 0.575	1.70 ± 0.710	1.667	0.096
Irritability/emotional instability	1.81 ± 0.564	1.72 ± 0.880	1.064	0.287
Abnormal motor behavior	1.82 ± 0.575	1.70 ± 0.710	1.667	0.096
Sleep/night behavior	1.80 ± 0.585	1.69 ± 0.713	1.555	0.120
Appetite and eating disorders	1.82 ± 0.575	1.71 ± 0.753	1.500	0.134

*p < 0.05.

Then we brought the above variables into Cox regression equation, and the results showed that male sex (p = 0.001, OR = 0.489, 95%CI: 0.319~0.751) and reading (p = 0.023, OR = 0.533, 95%CI: 0.310~0.917) were the protective factors for the development of aMCI (**Table 2** shows the related results).

**Table 2:** Risk and protective factors for patients with aMCI.

Variables	B	S.E.	Wald Score	df	p	OR	The 95% CI of OR	
							Lower limit	Upper limit
Male	-0.715	0.219	10.672	1	0.001	0.489	0.319	0.751
Reading	-0.629	0.277	5.168	1	0.023	0.533	0.310	0.917

### Cognitive Characteristics of the Old Normal Adult Who Were Followed Up

Next, we compared the cognitive characteristics between the two groups of people. In order to eliminate the influence of age, gender, and education on cognitive function, for each eligible case, a control subject matched for age, gender and education had been randomly selected from the subjects with cognitively unimpaired. So 111 pairs of cognitively unimpaired aging people matched in gender, age, and education were selected to participate in the final assessment. And we found that the baseline raw scores of Normal- aMCI group on Digit Span, Auditory verbal learning tests, Associative learning test, Verbal fluency, Wechsler Adult Intelligence Scale (WAIS)-III Block Design, and Wechsler Adult Intelligence Scale (WAIS)-III picture completion were lower than those of Normal-normal group (p < 0.05), while there was no statistical differences (p > 0.05) in MMSE, MoCA, Visual identification test and Delayed memory between two groups (**Table 3**). One year later, we re-evaluated all subjects and found that the scores of MMSE, MOCA, Digit Span, Auditory verbal learning tests, Associative learning test, Visual identification test, Verbal fluency, Delayed memory, Wechsler Adult Intelligence Scale (WAIS)-III Block Design, and Wechsler Adult Intelligence Scale (WAIS)-III picture completion of Normal-aMCI group were significantly lower (p < 0.05) than those in the Normal-normal group (**Table 4**). By using Cox regression analysis and cluster analysis, (whether the subjects were converted to aMCI as a grouping variable and age as time variable, 111 pairs), we found that the baseline scores of Wechsler’s Block Design (p=0.027, OR=0.969, 95%CL 0.943~0.996) could predict whether subjects would turn into aMCI in the future (**Table 5**).

**Table 3:** Results of global cognitive functioning tests and neuropsychological tests in different cognitive domains in aMCI subjects and cognitively normal controls (baseline).

Neuropsychological test	Normal-aMCI (n = 111)	Normal-normal (n = 111)	t or Z	p-value
Age, y	75.30 ± 7.123	73.56 ± 8.623	1.511	0.133
Education, y	6.17 ± 5.550	7.36 ± 4.472	-1.671	0.096
Male, n (%)	37(33.3)	38(34.2)	0.020	1.000
Neuropsychological tests				
MMSE	25.28 ± 3.467	26.00 ± 4.439	-1.348	0.179
MoCA	20.13 ± 4.886	21.24 ± 6.011	-1.519	0.130
Digit Span	12.436 ± 4.165	14.266 ± 3.458	-3.535	<0.001*
Auditory verbal learning Test	35.596 ± 11.446	40.314 ± 15.044	-2.175	0.031*
Associative learning test	5.42 ± 3.821	6.87 ± 4.095	-2.629	0.009*
Visual identification test	15.00 ± 4.000	15.71 ± 4.483	-1.216	0.225
Verbal fluency	23.56 ± 7.874	26.88 ± 10.932	-2.530	0.012*
Delayed memory	15.50 ± 9.078	17.09 ± 10.139	-1.213	0.227
Wechsler’s picture completion	8.02 ± 3.391	9.10 ± 4.418	-2.209	0.044*
Wechsler’s Block Design	21.72 ± 8.142	24.50 ± 9.934	-2.251	0.025*

**Table 4:** Results of global cognitive functioning tests and neuropsychological tests in different cognitive domains in aMCI subjects and cognitively normal controls (follow-up).

Neuropsychological test	Normal-aMCI (n = 111)	Normal-normal (n = 111)	t or Z	p-value
MMSE	23.30 ± 4.753	25.81 ± 3.912	-4.302	<0.001*
MoCA	17.63 ± 5.702	21.80 ± 5.763	-5.421	<0.001*
Digit Span	11.873 ± 3.931	14.380 ± 3.874	-4.743	<0.001*
Auditory verbal learning Test	30.252 ± 13.039	37.476 ± 14.429	-3.770	<0.001*
Associative learning test	4.24 ± 4.021	6.18 ± 4.396	-3.312	0.001*
Visual identification test	12.93 ± 5.197	16.27 ± 4.350	-5.105	<0.001*
Verbal fluency	21.24 ± 9.321	27.13 ± 10.400	-4.315	<0.001*
Delayed memory	12.23 ± 9.236	16.44 ± 9.531	-3.198	0.002*
Wechsler’s picture completion	7.10 ± 3.880	9.72 ± 4.041	-4.874	<0.001*
Wechsler’s Block Design	20.50 ± 9.095	24.54 ± 9.885	-3.099	0.002*

**Table 5:** Neuropsychological tests that can be used to predict aMCI.

Variables	B	S.E	Wald	df	p	OR	95% CI
Wechsler’s Block Design	-0.031	0.014	4.917	1	0.027	0.969	0.943~0.996

## Discussion

In this study, we found that the prevalence of aMCI among the old adult in Chinese communities was 17.1%, similar to the prevalence found in Lima, Peru (the mean age of the 352 participants was 70.91 ± 7.07 years, three quarters (82.9%) were women, and the mean number of years of education was 11.9 ± 3.7 years) (32) and Tremembé, Brazil (this study included 630 individuals [mean age, 71.3 y (±7.99); mean years of education, 4.9 (±4.54); 397 (63.0%) were women]) (33). With the unification of diagnostic standards and tools for aMCI in the world, the prevalence of aMCI in different ethnic groups tends to be the same. And other studies conducted in China also showed that the prevalence of aMCI was between 14.2% and 22.3% (14, 34), so our findings were consistent. As far as I know, this is the first nationwide study involving the incidence of aMCI in China. And we found that the incidence of aMCI among individuals age 60 and over in 20 Chinese communities was 70.57 per 1,000 person-years, which was similar to what found in other studies , for example, Ravaglia G et al. (34) followed up 745 old adult Italian participants [mean age: 77.4 ± 8.0; 398(54.0%) were women, 256 (29.6%) had less than 3 years of education] without dementia for 4 years, and found 155 changed into MCI, so the incidence rate of MCI was 76.8 (95% CI = 66.8 - 88.4) per 1,000 person-years. However, Katz MJ (35) et al surveyed 1944 adults aged 70 or older (Bronx residents, 1168 dementia free at baseline; mean age, 78.8 y; average follow-up, 3.9 y), and found that the incidence of aMCI was 38/1000 person-years. And the disparity was likely due to investigation methods (our study only investigated the overall incidence of aMCI, but did not focus on age and gender specificity). What’s more, the lack of epidemiological studies in China and the potential over-diagnosis of this entity should also be considered (32).

Previous studies indicated that 35%–75% of patients with aMCI would experience at least one neuropsychiatric symptom (NPS), such as delusions, aggressiveness, anxiety, depression, aberrant motor behavior, and eating problems (36). And the present of NPS was proved to be associated with decreased quality of life, increased hospital stay, as well as decreased survival (37). What’s more, the presence of NPS among aMCI patients would also increase the risk of progression to dementia (38, 39). In the present study, we used NPI to investigate the NPS of normal old adult people, and found that the total and subtest scores of NPI in normal-aMCI group were significant higher than that in the normal-normal group, although the difference was not statistically significant (p > 0.05). Therefore, we speculated that mental symptoms would gradually increase with the deterioration of cognitive symptoms. However, due to the serious missing of data, we were unable to count the prevalence of NPS in the aMCI and AD population, which was also a major limitation of this study.

By using Cox regression analysis, we confirmed that male sex (p = 0.001, OR = 0.489, 95%CI 0.319~0.751) and reading (p = 0.023, OR = 0.533, 95%CI 0.310~0.917) were protective factors for against aMCI. Evidence from epidemiological studies has indicated a lower prevalence of AD in males than in age-matched females (40), independent of race, culture, and diagnostic criteria used (41). In populations over the age of 65, men are half as likely to suffer from AD as women (42). However, a meta- analysis of fifty-six studies also showed that there were no statistically significant sex differences in the prevalence or incidence of amnestic MCI (43). Therefore, a larger sample of longitudinal studies is needed to specifically explore the gender differences in aMCI. There are several mechanisms to explain the difference in AD prevalence between men and women, first, compared to female with AD, male AD patients tend to have a better score on a variety of neuropsychological tasks, greater hippocampal volumes, less total brain atrophy and temporal lobe degeneration (44). In our present study, we also found that the volume of left (3,945.76 ± 470.139) and right hippocampus(3,724.5 ± 452.619) in normal old men was significantly larger (p < 0.001) than that(left hippocampus:3,671.84 ± 474.656; right hippocampus:3,504.96 ± 383.105) in women; second, estrogens can affect the prevalence of AD in women, but not in men (45); third, women often live longer than men, since age is the strongest risk factor for sporadic AD (46), as a result, women have a higher risk of AD; Fourth, risk factors (e.g., apolipoprotein E [APOE] genotype, type 2 diabetes, metabolic syndrome, obesity) that are equally common in women and men but are more common or have a stronger effect in one sex or gender group (46). Fifth, females often show higher frontal cortex cholinergic activity whereas males have higher activity in the hippocampus (47).

In addition, we also found that reading was an important factor for preventing aMCI, which was consistent with the findings of Wang YP (the research method is similar to ours) (48). So our conclusions were consistent. It is worth noting that there was a low rate of illiteracy in the present study, since people with higher socioeconomic status/education may also be more likely to read (49), the relationship between reading and aMCI needs further study.

Next, we explored the cognitive characteristics of people who would develop into aMCI in the future. And found that the old adult who would develop into aMCI in the future would show multiple cognitive impairments (such as immediate memory, associative learning memory, and executive function) in the early stage, although their overall cognitive function was not significantly abnormal. Then, we further explored which neuropsychological test can predict the transition from normal aging to aMCI. By using Cox regression analysis and cluster analysis, (whether the subjects were converted to aMCI as a grouping variable and age as time variable, 111 pairs), we found that Wechsler’s Block Design (p = 0.027, OR = 0.969, 95%CL 0.943~0.996) could predict whether subjects would turn into aMCI in the fucture. Although the impairment of memory is regarded as a hallmark of aMCI, recent studies (50, 51) have demonstrated that executive dysfunction may also be present. And Johns et al. (51) found that aMCI would show deficits in at least one sub-domain of executive function, independent of whether they were of a single domain or multiple domain subtype. Therefore, we inferred that executive dysfunction might be an indicator of future transition to aMCI, which was harmonious with Chapman’s findings (they also found that speed executive functioning was a strong predictor of MCI conversion to AD) (52).

## Limitations

We have to admit that there are some limitations in our research. First, we did not specifically discuss the relationship between the different subtypes of aMCI and cognitive function. Second, longitudinal data from larger samples was needed to verify the above conclusions. Third, although we also collected the social activities and exchange information of the participants, we did not include them in the final statistics due to the serious lack of data. Fourth, neuropsychiatric symptoms were a common accompaniment of dementia or aMCI (53), however, we did not concentrate on them in this study.

## Conclusions

In summary, using the nationwide community-based data, we found out that the prevalence and incidence aMCI among the old adult in Chinese communities was 17.1% and 70.57 per 1,000 person-years, respectively. And male sex and reading are protective factors for against aMCI. In addition, the old adult who will develop aMCI in the future will show multiple cognitive impairments (such as immediate memory, associative learning memory and executive function) in the early stage, and executive dysfunction might be an indicator of future transition to aMCI.

## Data Availability Statement

The datasets analyzed in this article are not publicly available. Requests to access the datasets should be directed to xiaoshifu@msn.com.

## Ethics Statement

The studies involving human participants were reviewed and approved by Shanghai Mental Health Centre. The patients/participants provided their written informed consent to participate in this study. Written informed consent was obtained from the individual(s) for the publication of any potentially identifiable images or data included in this article.

## Author Contributions

WL wrote this article. LS analyzed the data. SX was the project leader.

## Funding

This work was supported by grants from the Shanghai Jiao Tong University Technological Innovation Special Fund (YG2016MS38), the Cultivation of Multidisciplinary Interdisciplinary Project in Shanghai Jiaotong University (YG2019QNA10), and the Shanghai Mental Health Center Clinical Research Center (CRC2017ZD02).

## Conflict of Interest

The authors declare that the research was conducted in the absence of any commercial or financial relationships that could be construed as a potential conflict of interest.

## Supplementary Material

Table S1 Demographic, health, and disease-related for the follow up and lost population.

## References

[1] Xiao S Li J Tang M Chen W Bao F Wang H Methodology of China's national study on the evaluation, early recognition, and treatment of psychological problems in the elderly: the China Longitudinal Aging Study (CLAS). Shanghai Arch Psychiatry (2013) 25(2):91–8. 10.3969/j.issn.1002-0829.2013.02.005 PMC4054537 24991140

[2] Albert MS DeKosky ST Dickson D Dubois B Feldman HH Fox NC The diagnosis of mild cognitive impairment due to Alzheimer's disease: recommendations from the National Institute on Aging-Alzheimer's Association workgroups on diagnostic guidelines for Alzheimer's disease. Alzheimer's Dement (2011) 7(3):270–9. 10.1016/j.jalz.2011.03.008 PMC3312027 21514249

[3] Petersen RC Morris JC Mild cognitive impairment as a clinical entity and treatment target. Arch Neurol (2005) 62(7):1160–3. 10.1001/archneur.62.7.1160 16009779

[4] De Simone MS Perri R Fadda L Caltagirone C Carlesimo GA Predicting progression to Alzheimer's disease in subjects with amnestic mild cognitive impairment using performance on recall and recognition tests. J Neurol (2019) 266(1):102–11. 10.1007/s00415-018-9108-0 30386876

[5] Petersen RC Parisi JE Dickson DW Johnson KA Knopman DS Boeve BF Neuropathologic features of amnestic mild cognitive impairment. Arch Neurol (2006) May 63(5):665–72. 10.1001/archneur.63.5.665 16682536

[6] Petersen RC Stevens JC Ganguli M Tangalos EG Cummings JL DeKosky ST Practice parameter: early detection of dementia: mild cognitive impairment (an evidence-based review). Report of the Quality Standards Subcommittee of the American Academy of Neurology. Neurology (2001) 56(9):1133–42. 10.1212/WNL.56.9.1133 11342677

[7] Zhao Z Lu J Jia X Chao W Han Y Jia J Selective changes of resting-state brain oscillations in aMCI: an fMRI study using ALFF. Biomed. Res. Int. (2014) 2014:92–108. 10.1155/2014/920902 PMC4005061 24822220

[8] Ward A Arrighi HM Michels S Cedarbaum JM Mild cognitive impairment: disparity of incidence and prevalence estimates. Alzheimer's Dement (2012) 8(1):14–21. 10.1016/j.jalz.2011.01.002 22265588

[9] Cid-Fernandez S Lindin M Diaz F The importance of age in the search for ERP biomarkers of aMCI. Biol Psychol (2019) 142:108–15. 10.1016/j.biopsycho.2019.01.015 30721717

[10] Hao L Wang X Zhang L Xing Y Guo Q Hu X Prevalence, risk factors, and complaints screening tool exploration of subjective cognitive decline in a large cohort of the Chinese population. J Alzheimer's Dis (2017) 60(2):371–88. 10.3233/JAD-170347 28869471

[11] Ma F Wu T Zhao J Ji L Song A Zhang M Prevalence of mild cognitive impairment and its subtypes among Chinese older adults: role of vascular risk factors. Dement Geriatric Cogn Disord (2016) 41(5-6):261–72. 10.1159/000446507 27304680

[12] Wajman JR Mansur LL Yassuda MS Lifestyle patterns as a modifiable risk factor for late-life cognitive decline: a narrative review regarding dementia prevention. Curr Aging Sci (2018) 11(2):90–9. 10.2174/1874609811666181003160225 30280679

[13] Xue J Li J Liang J Chen S The prevalence of mild cognitive impairment in china: a systematic review. Aging Dis (2018) 9(4):706–15. 10.14336/AD.2017.0928 PMC6065290 30090658

[14] Hu C Yu D Sun X Zhang M Wang L Qin H The prevalence and progression of mild cognitive impairment among clinic and community populations: a systematic review and meta-analysis. Int Psychogeriatrics (2017) 29(10):1595–608. 10.1017/S1041610217000473 28884657

[15] Su X Shang L Xu Q Li N Chen J Zhang L Prevalence and predictors of mild cognitive impairment in Xi'an: a community-based study among the elders. PLoS One (2014) 9(1):657–75. 10.1371/journal.pone.0083217 PMC3885430 24421876

[16] Petersen RC Doody R Kurz A Mohs RC Morris JC Rabins PV Current concepts in mild cognitive impairment. Arch Neurol (2001) 58(12):1985–92. 10.1001/archneur.58.12.1985 11735772

[17] Petersen RC Caracciolo B Brayne C Gauthier S Jelic V Fratiglioni L Mild cognitive impairment: a concept in evolution. J Internal Med (2014) 275(3):214–28. 10.1111/joim.12190 PMC3967548 24605806

[18] Abdulrab K Heun R Subjective memory impairment. A review of its definitions indicates the need for a comprehensive set of standardised and validated criteria. Eur Psychiatry (2008) 23(5):321–30. 10.1016/j.eurpsy.2008.02.004 18434102

[19] Reisberg B Diagnostic criteria in dementia: a comparison of current criteria, research challenges, and implications for DSM-V. J Geriatric Psychiatry Neurol (2006) 19(3):137–46. 10.1177/0891988706291083 16880355

[20] McKhann G Drachman D Folstein M Katzman R Price D Stadlan EM Clinical diagnosis of Alzheimer's disease: report of the NINCDS-ADRDA Work Group under the auspices of Department of Health and Human Services Task Force on Alzheimer's Disease. Neurology (1984) 34(7):939–44. 10.1212/WNL.34.7.939 6610841

[21] Roman GC Tatemichi TK Erkinjuntti T Cummings JL Masdeu JC Garcia JH Vascular dementia: diagnostic criteria for research studies. Report of the NINDS-AIREN International Workshop. Neurology (1993) 43(2):250–60. 10.1212/WNL.43.2.250 8094895

[22] Morris JC The Clinical Dementia Rating (CDR): current version and scoring rules. Neurology (1993) 43(11):2412–4. 10.1212/WNL.43.11.2412-a 8232972

[23] Loewenstein DA Curiel RE Wright C Sun X Alperin N Crocco E Recovery from proactive semantic interference in mild cognitive impairment and normal aging: relationship to atrophy in brain regions vulnerable to alzheimer's disease. J Alzheimer's Dis (2017) 56(3):1119–26. 10.3233/JAD-160881 PMC5660921 28106554

[24] O'Bryant S Humphreys JG Ivnik R Graff-Radford N Petersen R Lucas J Detecting dementia with the mini-mental state examination in highly educated individuals. Arch Neurol (2008) 65(7):963–7. 10.1001/archneur.65.7.963 PMC2587038 18625866

[25] Gil L DSC R Gil F Romero SJ Pretelt BF Validation of the Montreal Cognitive Assessment (MoCA) in Spanish as a screening tool for mild cognitive impairment and mild dementia in patients over 65 years old in Bogotá, Colombia. Int J Geriatric Psychiatry (2014) 30(6):655–63. 10.1002/gps.4199 25320026

[26] Leung JLM Lee GTH Lam YH Chan RCC Wu JYM The use of the Digit Span Test in screening for cognitive impairment in acute medical inpatients. Int Psychogeriatrics (2011) 23(10):1569–74. 10.1017/S1041610211000792 21729426

[27] Xia H Zhang ZX Li-Yong WU Shi LL Zhao XH Validity of auditory verbal learning test in diagnosis of alzheimer's disease. Acta Academiae Med Sinicae (2012) 34(3):262–6. 10.3881/j.issn.1000-503X.2012.03.014 22776661

[28] Cummings JL Mega M Gray K Rosenberg-Thompson S Carusi DA Gornbein J The neuropsychiatric inventory: comprehensive assessment of psychopathology in dementia. Neurology (1994) 44(12):2308–14. 10.1212/WNL.44.12.2308 7991117

[29] Wang T Xiao S Chen K Yang C Dong S Cheng Y Prevalence, incidence, risk and protective factors of amnestic mild cognitive impairment in the elderly in Shanghai. Curr Alzheimer Res (2017) 14(4):460–6. 10.2174/1567205013666161122094208 27875948

[30] Wolz R Schwarz AJ Yu P Cole PE Rueckert D Jack CR Robustness of automated hippocampal volumetry across magnetic resonance field strengths and repeat images. Alzheimer's Dement (2014) 10(4):430–438.e432. 24985688 10.1016/j.jalz.2013.09.014

[31] Mao C Li ZH Lv YB Cao X Zhou B KR B Specific leisure activities and cognitive functions among the oldest-old: the Chinese longitudinal healthy longevity survey. J Gerontol Ser A Biol Sci Med Sci (2019). 474:99–108. 10.1093/gerona/glz086 PMC6776703 30946444

[32] Sanchez SS Abanto J Frequency and associated factors of amnestic mild cognitive impairment at four senior citizen clubs in Lima, Peru. (2019). Dement. Neuropsychol. 13: (3):321–8. 10.1590/1980-57642018dn13-030009 PMC6753901 31555405

[33] Cesar KG Brucki SM Takada LT Nascimento LF Gomes CM Almeida MC Prevalence of cognitive impairment without dementia and dementia in Tremembe, Brazil. Alzheimer Dis Assoc Disord (2016) 30(3):264–71. 10.1097/WAD.0000000000000122 26629676

[34] Rao D Luo X Tang M Prevalence of mild cognitive impairment and its subtypes in community-dwelling residents aged 65 years or older in Guangzhou, China. Arch Gerontol Geriatrics (2018) 75:70–5. 10.1016/j.archger.2017.11.003 29197258

[35] Katz MJ Lipton RB Hall CB Zimmerman ME Sanders AE Verghese J Age-specific and sex-specific prevalence and incidence of mild cognitive impairment, dementia, and Alzheimer dementia in blacks and whites: a report from the Einstein Aging Study. Alzheimer Dis Assoc Disord (2012) 26(4):335–43. 10.1097/WAD.0b013e31823dbcfc PMC3334445 22156756

[36] Apostolova LG Cummings JL Neuropsychiatric manifestations in mild cognitive impairment: a systematic review of the literature. Dementia Geriatric Cogn Disord (2008) 25(2):115–26. 10.1159/000112509 18087152

[37] Kazui H Yoshiyama K Kanemoto H Suzuki Y Sato S Hashimoto M Differences of behavioral and psychological symptoms of dementia in disease severity in four major dementias. PLoS One (2016) 11(8):92–103. 10.1371/journal.pone.0161092 PMC4990196 27536962

[38] Rosenberg PB Mielke MM Appleby BS Oh ES Geda YE Lyketsos CG The association of neuropsychiatric symptoms in MCI with incident dementia and Alzheimer disease. Am J Geriatric Psychiatry (2013) 21(7):685–95. 10.1016/j.jagp.2013.01.006 PMC3428504 23567400

[39] Forrester SN Gallo JJ Smith GS Leoutsakos JM Patterns of neuropsychiatric symptoms in mild cognitive impairment and risk of dementia. Am J Geriatric Psychiatry (2016) 24(2):117–25. 10.1016/j.jagp.2015.05.007 PMC4646727 26209222

[40] Mielke MM Vemuri P Rocca WA Clinical epidemiology of Alzheimer's disease: assessing sex and gender differences. Clin Epidemiol (2014) 6:37–48. 10.2147/CLEP.S37929 24470773 PMC3891487

[41] Baron S Ulstein I Werheid K Psychosocial interventions in Alzheimer's disease and amnestic mild cognitive impairment: evidence for gender bias in clinical trials. Aging Ment Health (2015) 19(4):290–305. 10.1080/13607863.2014.938601 25048626

[42] Hy LX Keller DM Prevalence of AD among whites: a summary by levels of severity. Neurology (2000) 55(2):198–204. 10.1212/WNL.55.2.198 10908890

[43] Au B Dale-McGrath S Tierney MC Sex differences in the prevalence and incidence of mild cognitive impairment: a meta-analysis. Ageing Res Rev (2017) 35:176–99. 10.1016/j.arr.2016.09.005 27771474

[44] Li W Qiu Q Sun L Yue L Wang T Li X Sex differences in obesity and cognitive function in a cognitively normal aging Chinese Han population. Neuropsychiatr Dis Treat (2017) 13:2405–10. 10.2147/NDT.S145245 PMC5604567 29066899

[45] Pike CJ Sex and the development of Alzheimer's disease. J Neurosci Res (2017) 95(1-2):671–80. 10.1002/jnr.23827 PMC5120614 27870425

[46] Nebel RA Aggarwal NT Barnes LL Gallagher A Goldstein JM Kantarci K Understanding the impact of sex and gender in Alzheimer's disease: a call to action. Alzheimer's Dement (2018) 14(9):1171–83. 10.1016/j.jalz.2018.04.008 PMC6400070 29907423

[47] Giacobini E Pepeu G Sex and gender differences in the brain cholinergic system and in the response to therapy of Alzheimer disease with cholinesterase inhibitors. Curr Alzheimer Res (2018) 15(11):1077–84. 10.2174/1567205015666180613111504 29895246

[48] Wang YP Zhai JB Zhu F Zhang WW Yang XJ Qu CY A three-year follow-up study on the transfer of mild cognitive impairment to Alzheimer's disease among the elderly in Taiyuan city. Zhonghua Liu Xing Bing Xue za zhi = Zhonghua Liuxingbingxue zazhi (2011) 32(2):105–9. 21518614

[49] Romeo RR Christodoulou JA Halverson KK Murtagh J Cyr AB Schimmel C Socioeconomic status and reading disability: neuroanatomy and plasticity in response to intervention. Cereb Cortex (N Y N.Y.: 1991). (2018) 28(7):2297–312. 10.1093/cercor/bhx131 PMC5998958 28591795

[50] Brandt J Aretouli E Neijstrom E Selectivity of executive function deficits in mild cognitive impairment. Neuropsychology (2009) 23(5):607–18. 10.1037/a0015851 PMC2769993 19702414

[51] Johns EK Phillips NA Belleville S The profile of executive functioning in amnestic mild cognitive impairment: disproportionate deficits in inhibitory control. J Int Neuropsychol Soc (2012) 18(3):541–55. 10.1017/S1355617712000069 22370245

[52] Chapman RM Mapstone M McCrary JW Gardner MN Porsteinsson S Sandoval TC Predicting conversion from mild cognitive impairment to Alzheimer's disease using neuropsychological tests and multivariate methods. J Clin Exp Neuropsychol (2011) 33(2):187–99. 10.1080/13803395.2010.499356 PMC3048956 20711906

[53] Kales HC Gitlin LN Lyketsos CG Assessment and management of behavioral and psychological symptoms of dementia. BMJ (Clin Res ed.) (2015) 350–69. 10.1136/bmj.h369 PMC4707529 25731881

