# Supplementary material for: Prevalence, Incidence, Influence Factors, and Cognitive Characteristics of Amnestic Mild Cognitive Impairment Among Older Adult: A 1-Year Follow-Up Study in China
Source: Front Psychiatry. 2020 Feb 28;11:75. doi: 10.3389/fpsyt.2020.00075 (PMC7058542; doi:10.3389/fpsyt.2020.00075)
Supplement: Table S1 — Demographic, health, and disease-related for the follow up and lost population. [file Table_1.docx]

| Characteristics | Follow up  (N=1573) | Lost  (n=645) | t/ X^2^ | P-value |
| --- | --- | --- | --- | --- |
| Age, y | 70.44±7.572 | 69.50±7.422 | 2.595 | 0.010* |
| Education, y | 9.39±5.09 | 8.92±7.008 | 1.731 | 0.084 |
| Male,n (%) | 765(48.6) | 309(47.9) | 0.097 | 0.779 |
| Smoking,n (%) | 449(28.5) | 200(31.2) | 1.498 | 0.237 |
| Drinking alcohol,n (%) | 314(20.0) | 161(25.3) | 7.584 | 0.007* |
| Drinking tea,n (%) | 805(51.2) | 313(48.5) | 1.284 | 0.262 |
| Taking exercise,n (%) | 1186(75.4) | 502(78.7) | 2.714 | 0.109 |
| Hobby,n (%) | 1013(64.4) | 296(47.7) | 51.806 | <0.001* |
| Reading,n (%) | 514(32.7) | 131(32.4) | 0.014 | 0.953 |
| Playing music, n (%) | 394(25.1) | 108(27.9) | 1.317 | 0.269 |
| Surfing the internet, n (%) | 180(11.4) | 38(7.8) | 5.095 | 0.023* |
| Depression , n (%) | 74(4.7) | 27(4.2) | 0.283 | 0.655 |
| Diabetes, n (%) | 232(14.8) | 114(18.5) | 7.161 | 0.028 |
| Hypertension, n (%) | 746(47.4) | 283(43.9) | 2.317 | 0.134 |

Table S1 - Demographic, health, and disease-related for the follow up and lost population
